# Transcriptome differences in adipose stromal cells derived from pre- and postmenopausal women

**DOI:** 10.1186/s13287-020-01613-x

**Published:** 2020-02-28

**Authors:** Yun Xie, Bin Fang, Wenhui Liu, Guangshuai Li, Ru-Lin Huang, Lu Zhang, Jiahao He, Shuangbai Zhou, Kai Liu, Qingfeng Li

**Affiliations:** 1 Department of Plastic and Reconstructive Surgery, Shanghai Ninth People’s Hospital, Shanghai Jiao Tong University School of Medicine, 639 Zhizaoju Road, Shanghai, 200011 China; 2 Plastic & Reconstructive Surgery of the First Affiliated Hospital of Zhengzhou University, 1 Jianshe East Road, Zhengzhou, 450052 China

**Keywords:** Adipose stromal cells, Premenopausal, Postmenopausal, Transcriptome, Immunoregulation

## Abstract

**Background:**

As the population ages, an increasing number of postmenopausal women are donors of adipose stromal cells (ASCs) and may benefit from autologous ASC-related treatments. However, the effect of menopausal status on ASCs has not been investigated.

**Methods:**

RNA sequencing data were downloaded, and differentially expressed genes (DEGs) were identified. Hierarchical clustering, Gene Ontology, and pathway analyses were applied to the DEGs. Two gene coexpression network analysis approaches were applied to the DEGs to provide a holistic view and preserve gene interactions. Hub genes of the gene coexpression network were identified, and their expression profiles were examined with clinical samples. ASCs from pre- and postmenopausal women were co-cultured with monocytes and T cells to determine their immunoregulatory role.

**Results:**

In total, 2299 DEGs were identified and presented distinct expression profiles between pre- and postmenopausal women. Gene Ontology and pathway analyses revealed some fertility-, sex hormone-, immune-, aging-, and angiogenesis-related terms and pathways. Gene coexpression networks were constructed, and the top hub genes, including TIE1, ANGPT2, RNASE1, PLVAP, CA2, and MPZL2, were consistent between the two approaches. Expression profiles of hub genes from the RNA sequencing data and clinical samples were consistent. ASCs from postmenopausal women elicit M1 polarization, while their counterparts facilitate CD3/4+ T cell proliferation.

**Conclusions:**

The present study reveals the transcriptome differences in ASCs derived from pre- and postmenopausal women and provides holistic views by preserving gene interactions via gene coexpression network analysis. The top hub genes identified by this study could serve as potential targets to enhance the therapeutic potential of ASCs.

## Background

Adipose stromal cells (ASCs) are a group of mesenchymal stromal cells routinely isolated from the stromal vascular fraction of adipose tissue. In addition to preserving the main characteristics of mesenchymal stromal cells, ASCs are also distinguished by their high abundance and easy access [1], which makes them very promising candidates in numerous clinical trials targeting cardiac, immunological, rheumatological, and many other diseases [2]. One frequently used method to obtain ASCs is liposuction [3, 4]. It has been shown that donor traits such as age, body mass index, gender, donor site, and menopausal status impact ASC viability and function during liposuction [5]. Among these factors, menopausal status may be an important characteristic that interacts with aging, hormonal status, and many other factors. However, the effects of menopausal status on ASCs have not been thoroughly investigated.

Some studies provide an indirect profile of the relationship between menopausal status and ASCs by focusing on aging and sex hormones. Aging has been reported to impair the proliferation, differentiation, and angiogenic capacity of ASCs [6–9]. Estrogen has long been recognized as able to regulate ASC proliferation, migration, and differentiation [10], while ASCs have been shown to affect ovary function. Sun et al. found that topical or systematical application of ASCs could improve mouse ovary function in a chemotherapy-induced ovary failure model [11]. Unlike ASCs, the relationship between bone marrow mesenchymal stromal cells and menopausal status has been preliminarily investigated by Liu and his colleagues [12]. They found that bone marrow mesenchymal stromal cells derived from pre- and postmenopausal rats have different characteristics. These studies are very inspiring, as direct data focused on the effects of menopausal status on ASCs are still lacking.

As the population ages, an increasing number of postmenopausal women may desire liposuction, which is one of the most important sources of ASCs. These women may also benefit from autologous ASC-related treatments. Thus, we need to determine the effect of menopausal status on ASCs. The present study focuses on the transcriptome differences associated with ASCs derived from pre- and postmenopausal women and annotates the changes with integrated bioinformatics approaches. We also applied Weighted Gene Coexpression Network Analysis (WGCNA) and Multiscale Embedded Gene Coexpression Network Analysis (MEGENA) to reveal gene interaction networks and identify the key players in ASCs pre- and postmenopause. These hub genes may serve as targets to enhance the therapeutic potential of ASCs. The present study can support better applications and update our perspectives of ASCs, especially in postmenopausal women.

## Materials and methods

### Data acquisition and raw data processing

RNA sequencing data were downloaded from the National Center for Biotechnology Information (NCBI) Gene Expression Omnibus [13] under accession number GSE86244, which was provided by Shan et al. [14]. We only selected ASC data from women < 45 years old (12 patients in the premenopause group) and > 55 years old (3 patients in the postmenopause group). All ASCs were isolated from adipose tissue harvested during elective abdominoplasty. Raw data were transformed with the fastq-dump software included in the NCBI SRA Toolkit (www.ncbi.nlm.nih.gov/sra). Quality control was performed with FastQC (www.bioinformatics.babraham.ac.uk/projects/fastqc/), and the data quality looked quite good. Thus, we skipped the data trimming process. The reads were then aligned against the GRCh387 reference genome downloaded from Ensembl [15] with hisat2 [16]. Transcripts were assembled with StringTie [17], and differentially expressed genes (DEGs) were identified by DESeq2 [18] by comparing the genes in postmenopausal women to premenopausal women. DEGs were determined as those with a *p* value less than 0.05 and no further filtering with an adjusted *p* value and fold change to keep potential meaningful genes as much as possible at the cost of increasing the computational burden of downstream analysis.

### Annotation of DEGs

To provide an overview of the DEGs, we applied hierarchical clustering analysis using MeV [19]. MeV generated a dendrogram aligned with a heat map that could be used to check whether DEGs were present in distinct expression profiles between pre- and postmenopausal women.

To provide a complete picture of the changes associated with menopause, we applied Gene Ontology (GO) and pathway analyses to annotate the DEGs. GO includes three structured ontologies that describe gene products in terms of their associated biological processes, cellular components, and molecular functions in a species-independent manner [20, 21]. GO analysis is an enrichment analysis that determines which GO terms are overrepresented or underrepresented for a given set of genes. Upregulated and downregulated genes were analyzed separately. GO analysis can be performed by many means; we chose GOSlimViewer [22] to provide a high-level summary of the GO terms using a Generic GO slim set. According to the Gene Ontology Consortium, GO slims are filtered versions of GO ontologies that contain a subset of the terms from the complete GO and offer a broad overview of the ontology content.

Pathway analysis was achieved with the Database for Annotation, Visualization and Integrated Discovery (DAVID, http://david.abcc.ncifcrf.gov/) [23] by interrogating the Kyoto Encyclopedia of Genes and Genomes (KEGG) database [24]. Upregulated and downregulated genes were analyzed separately. KEGG is a canonical pathway analysis method and is well recognized by the scientific community. However, as reviewed in [25], KEGG, as an over-representation analysis, only considers the number of genes alone and ignores any values associated with them. Thus, the DEG information was not fully exploited. We then used the Ingenuity Pathway Analysis Database (IPA, www.Ingenuity.com), which considers the DEG fold changes and *p* values as a supplement, and all the DEGs were analyzed together. The combination of KEGG and IPA provided integrated and precise pathway analysis results for the DEGs.

### Gene coexpression network analysis

After identification of the DEGs, a common procedure is the double filtration of DEGs based on their fold change and *p* value [26]. Highly ranked DEGs could serve important biological functions and are usually selected for downstream analysis. However, biological processes are usually cascade-like. Thus, downstream genes usually rank higher than upstream genes when applying double filtration, and gene interactions are often neglected when focusing on individual DEGs alone. Therefore, we also applied WGCNA and MEGENA to reveal gene interaction networks and identify the key players in ASCs pre- and postmenopause. To reduce the noise and preserve the main fluctuations in the genes, only DEGs were used in the gene coexpression network analysis; this was the basic and very first analysis step in the transcriptomic analysis procedure [27].

WGCNA was performed with the WGCNA package [28] in the static programming language and environment R [29]. WGCNA is distinguished as embracing the idea that gene networks follow a scale-free distribution pattern similar to many other biological networks [30]. First, a gene correlation matrix was transformed into a scale-free network. Second, a dynamic tree-cutting algorithm [31] was applied to perform unsupervised hierarchical clustering, and the clustering tree branches were defined as modules. Third, correlations between the modules and menopausal status were calculated, and a highly related module was selected. Module eigengenes, which are principal components of gene expression profiles, were selected as representatives for each module in this step, and menopausal status was defined as a binary variable. Fourth, hub genes in the selected module were sorted by ranking their intra-modular connectivity and correlation with the module eigengenes. Hub genes are the backbones of scale-free networks [32] and have been found to be biologically meaningful in many diseases [33–35].

MEGENA was performed with the MEGENA package [36] in R. Unlike WGCNA, MEGENA preserves gene interaction by beginning with Fast Planar Filtered Network Construction. Genes in this Planar Filtered Network (PFN) were then clustered by Multiscale Clustering Analysis. Hub genes from each cluster at each scale were then identified by Multiscale Hub Analysis. The developers of MEGENA suggest that the multiscale hub genes tend to be biologically meaningful [36]. MEGENA revealed meaningful multi-scale organizations of gene coexpression networks, which could be a good supplement to WGCNA. The hub genes identified by these two methods were also compared. We applied Gephi [37] to visualize the Weighted Gene Coexpression Network (WGCN) and Multiscale Embedded Gene Coexpression Network (MEGCN) with a Fruchterman-Reingold layout.

To check whether the selected module in WGCN and the multiscale hub genes in MEGCN are highly related to menopausal factors, we also annotated these genes. GO analysis, like KEGG, is a kind of over-representation analysis method that only considers the number of genes and ignores their values [25]. Thus, up- and downregulated genes should not be analyzed together with GO analysis, as they represent opposite tendencies. We scrutinized these genes and found that they were composed of both up- and downregulated genes. Therefore, GO analysis was not applicable, and IPA was applied.

### Conformation of hub gene expression

This study was approved by the Institutional Ethics Committee of Shanghai Ninth People’s Hospital. ASCs were isolated from subcutaneous adipose tissues of ten healthy donors who underwent abdominal liposuction after providing informed consent. Among these clinical samples, five patients were premenopausal women with an average age of 27.3, and five patients were postmenopausal women with an average age of 73.8.

We also performed immunophenotypic analysis and multipotential induction of ASCs.

The expression of the top hub genes, including TIE1, ANGPT2, RNASE1, PLVAP, CA2, and MPZL2, was verified with clinical samples by real-time polymerase chain reaction (RT-PCR). The primers used in this study are shown in Supplementary File 7. The housekeeping gene GAPDH was used for normalization. Significant differences among groups were assessed using the two-tailed Student’s *t* test. *p*<0.05 was considered statistically significant. The results are presented as the mean ± S.D.

### Immunoregulatory role of ASCs from pre- and postmenopausal women

Many immune system-related GO terms and pathways were identified in the present study. Therefore, we co-cultured ASCs with the human leukemia monocytic cell lines THP-1 and CD3/4+ T cells to determine the immunoregulatory role of ASCs from pre- and postmenopausal women. The mRNA expression of iNOS and Arg-1 (markers for M1 and M2 macrophages) was analyzed in THP-1 cells to determine the polarization of macrophages. The Alamar Blue assay and cell cycle analysis were performed for CD3/4+ T cells to determine the proliferation difference.

## Results

### Characteristics and annotation of the DEGs

**Fig. 1:**
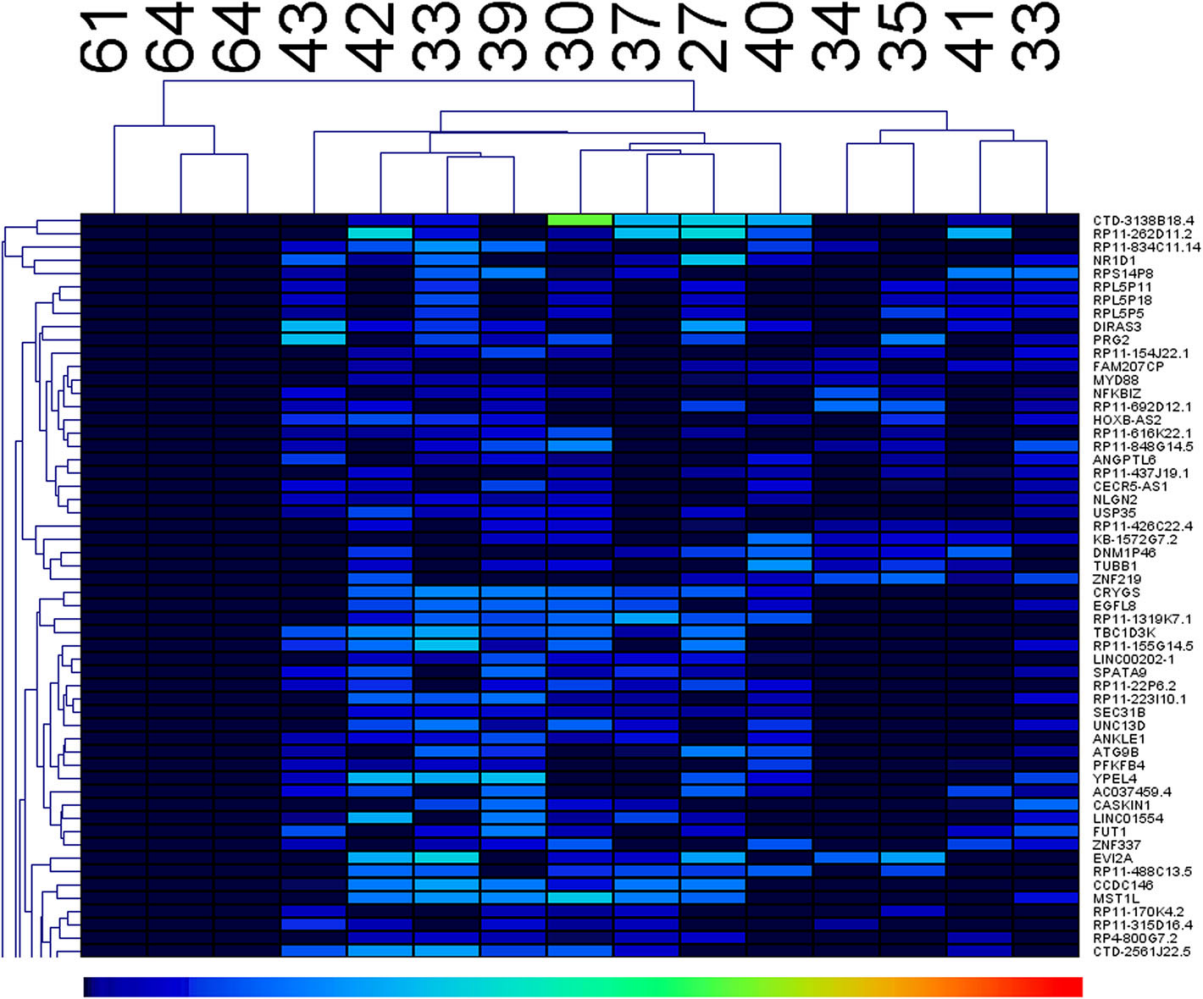
Hierarchical clustering analysis of the DEGs. A dendrogram of samples is provided on the top with the ages of corresponding samples given above the dendrogram. A dendrogram of genes is given on the left with the names of the corresponding genes on the right. The gene expression value scale is shown below the heatmap, with blue as the lowest and red as the highest

In total, 2299 genes were differentially expressed in the pre- and postmenopausal women. Among them, 794 genes were upregulated and 1505 genes were downregulated. A complete list of the identified genes is provided in Supplementary File 1. Hierarchical cluster analysis with MeV indicated that DEGs presented distinct expression profiles between pre- and postmenopausal women (Fig. 1). Pre- and postmenopausal women were clustered into two clusters. Only part of the hierarchical cluster results is shown for clarity, and the complete results are provided in Supplementary File 2.

GO analysis with GOSlimViewer revealed many basic biological processes that were altered pre- and postmenopause (Supplementary File 3), indicating that these DEGs participate in various biological processes. It is not surprising that the GO slim term Reproduction and Embryo Development was found for both up- and downregulated DEGs, indicating that the recession of fertility in postmenopausal women is reflected in ASCs. Two related terms, Anatomical Structure Development and Anatomical Structure Formation Involved in Morphogenesis, were also found. The slim term Ageing was also identified among the downregulated DEGs (Supplementary File 3). Immune System Process was found for both the up- and downregulated DEGs.

The pathway analysis results are shown in Table 1; only related pathways are listed, and the complete pathway analysis results are given in Supplementary File 4. The identification of oocyte meiosis, GNRH signaling, and relaxin signaling indicates that sex hormone- and fertility-related alterations are preserved among the DEGs to some extent. Immune and angiogenesis pathways were also identified.

**Table 1:** Pathway analysis of DEGs

Pathway	-log (*p* value)
KEGG-up	
Leukocyte transendothelial migration	3.40
Platelet activation	3.00
Oocyte meiosis	2.16
Vascular smooth muscle contraction	1.90
Endometrial cancer	1.37
Prostate cancer	1.36
IPA	
IL-8 signaling	2.22
Leukocyte extravasation signaling	1.70
TGF-β signaling	1.56
Inhibition of angiogenesis by TSP1	1.54
Nitric oxide signaling in the cardiovascular system	1.52
Corticotropin-releasing hormone signaling	1.49
GNRH signaling	1.47
CXCR4 signaling	1.43
Relaxin signaling	1.41

### Gene coexpression network analysis

**Fig. 2:**
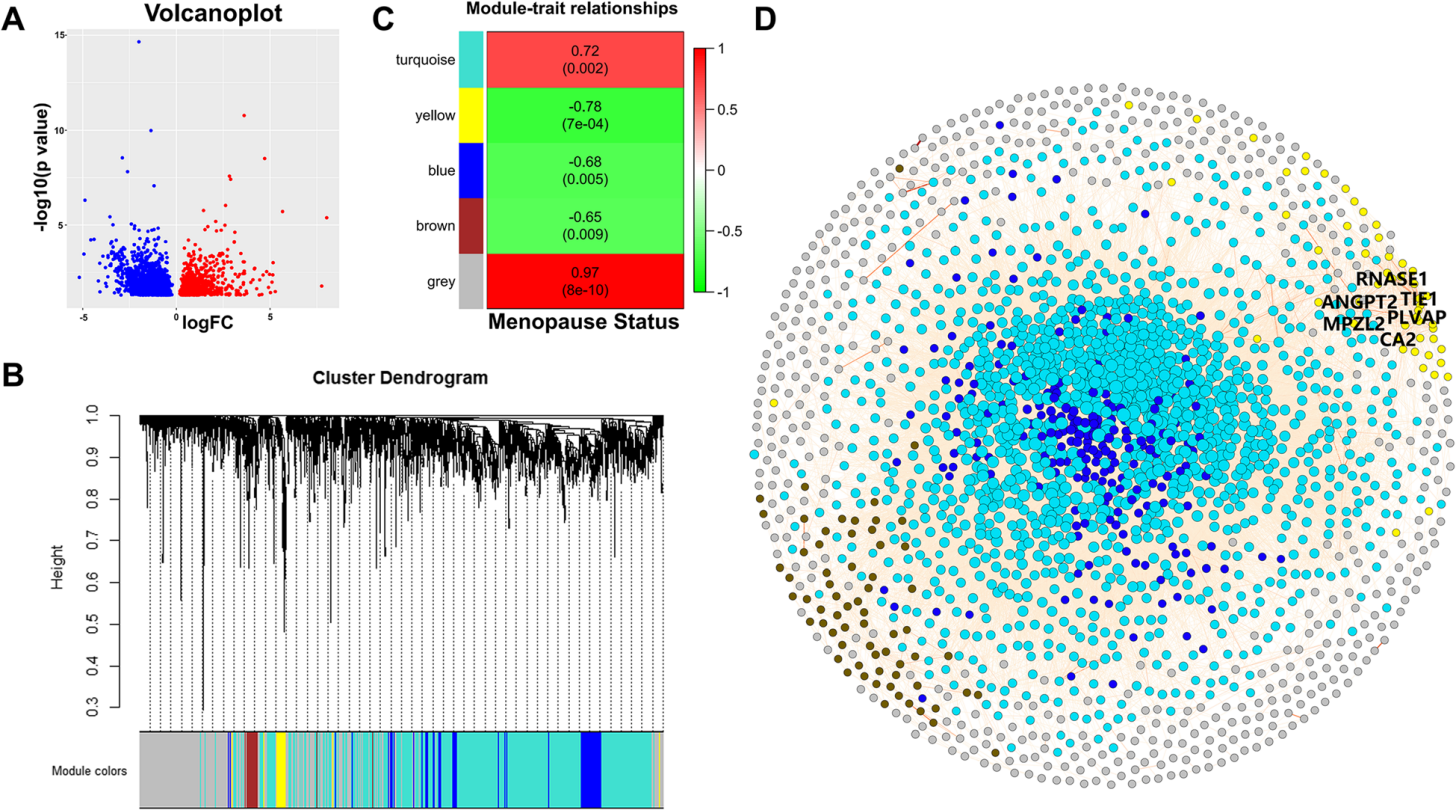
**a** Volcano plot of DEGs. The horizontal axis represents the logarithmic fold change, and the vertical axis shows the -log (*p* value) of the DEGs. **b** Hierarchical clustering of the WGCN. Each line in the hierarchical cluster tree represents a gene, and the distance between two genes in the WGCN is shown as the height on the vertical axis. The color bar beneath shows the module membership for each gene. **c** The module-trait relationships in the WGCN. Each cell represents a module with the module name and corresponding color at the left. The numbers near the cells are the correlation coefficient (upper) and corresponding *p* value (lower). **d** Visual representation of the WGCN. Each node represents a gene, and the color indicates module membership. Node size is proportional to the intra-modular connectivity. Edges between nodes represent interactions between genes, and darker edges indicate a higher correlation. The names of the top 6 hub genes are given in the yellow module

Canonical analysis methods focus on individual DEGs with high fold changes and a small *p* value, similar to those at the top left and right corners in the DEG volcano plot (Fig. 2a). However, a holistic view is not generated if we ignore interactions between genes. Thus, we applied WGCNA and MEGENA for these DEGs. WGCNA identified four modules within DEGs that we described as turquoise, yellow, blue, and brown (Fig. 2b). DEGs that could not be clustered into any specific modules were classified into a grey module. As shown in Fig. 2c, the yellow module is the most related to menopause status, with the highest absolute value correlation coefficient (− 0.78) and lowest *p* value (7e−04). The visual representation of the WGCN is shown in Fig. 2d, and a summary of the WGCN is provided in Supplementary File 5. As shown in Fig. 2d, genes belonging to the same module tend to be close to one another. The top 10 hub genes in the yellow module are TIE1, ANGPT2, PLVAP, CA2, RNASE1, MPZL2, SELE, SOX17, YBX1P6, and PPP1R16B, which were identified by ranking their intra-modular connectivity. Readers can refer to Supplementary File 5 for further details.

**Fig. 3:**
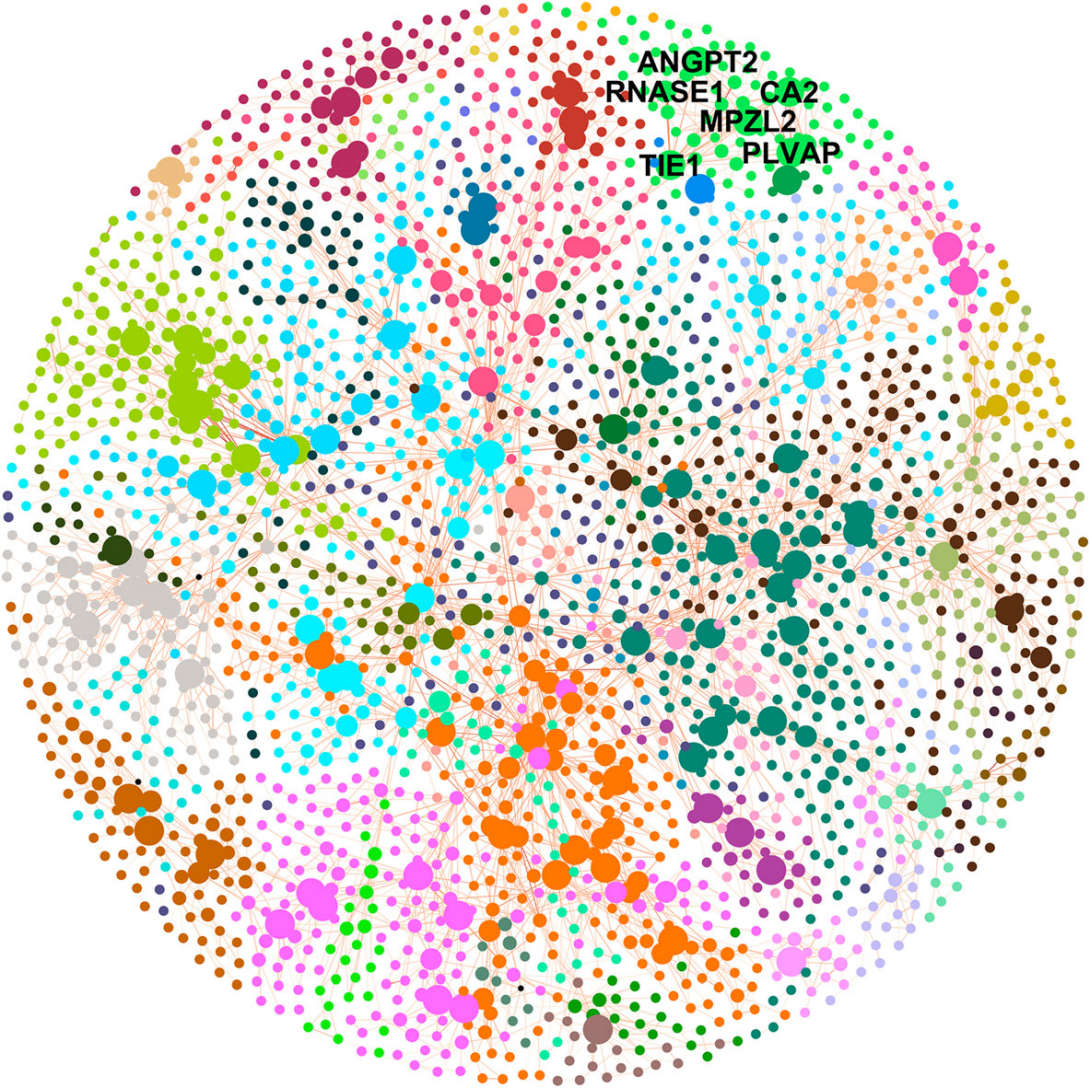
Multiscale Embedded Gene Coexpression Network. Each node represents a gene, and the color indicates the module membership. A gene may belong to more than one module on a different scale in MEGCN, and it would be colored according to the module on the highest scale in that case. Node size is proportional to its frequency in the three scales, i.e., hub genes in three scales are the largest and non-hub genes are the smallest. The names of some multi-scale hub genes are given

MEGENA identified 83 modules and 427 hub genes using three scales. Among the hub genes, 87 were hub genes in all three scales. The visual representation of MEGCN is given in Fig. 3. As shown in Fig. 3, genes belonging to the same module are usually clustered together. The complete MEGCN is summarized in Supplementary File 6, including information about the multiscale hub genes. By comparing the top hub genes in the WGCN yellow module with the multiscale hub genes in MEGCN, we found that 5 of the top 6 hub genes in the yellow module were also hub genes in all three scales in MEGCN. These 5 hub genes are TIE1, ANGPT2, PLVAP, CA2, and MPZL2. RNASE1, the last of the top 6 hub genes, was a hub gene in two scales in MEGCN. This consistency of the hub genes in WGCNA and MEGENA could support their authenticity to some extent. The identification of hub genes with these two methods is quite consistent.

We also applied IPA to examine whether the yellow module and multiscale hub genes are highly related to menopausal status. Some highly related pathways are shown in Table 2. Most pathways are either immune- or angiogenesis-related. Sex hormone-related pathways were also identified, such as GNRH signaling, relaxin signaling, and androgen signaling. It should be noted that the *p* value for androgen signaling is slightly greater than 0.05.

**Table 2:** Ingenuity pathway analysis of the yellow module and multiscale hub genes

Pathway	-log (*p* value)
Yellow module	
Agranulocyte adhesion and diapedesis	4.15
Granulocyte adhesion and diapedesis	3.13
Th2 pathway	2.25
Wnt/β-catenin signaling	2.08
Th1 and Th2 activation pathway	2.00
IL-8 signaling	1.90
Angiopoietin signaling	1.81
VEGF family ligand-receptor interactions	1.70
VEGF signaling	1.60
Nitric oxide signaling in the cardiovascular system	1.54
PTEN signaling	1.46
Multiscale hub genes	
IL-8 signaling	3.30
GNRH signaling	2.90
Angiopoietin signaling	2.66
Cardiac β-adrenergic signaling	1.95
Relaxin signaling	1.83
CXCR4 signaling	1.76
Breast cancer regulation by Stathmin1	1.51
IL-1 signaling	1.42
Androgen signaling	1.28

### Conformation of hub gene expression

**Fig. 4:**
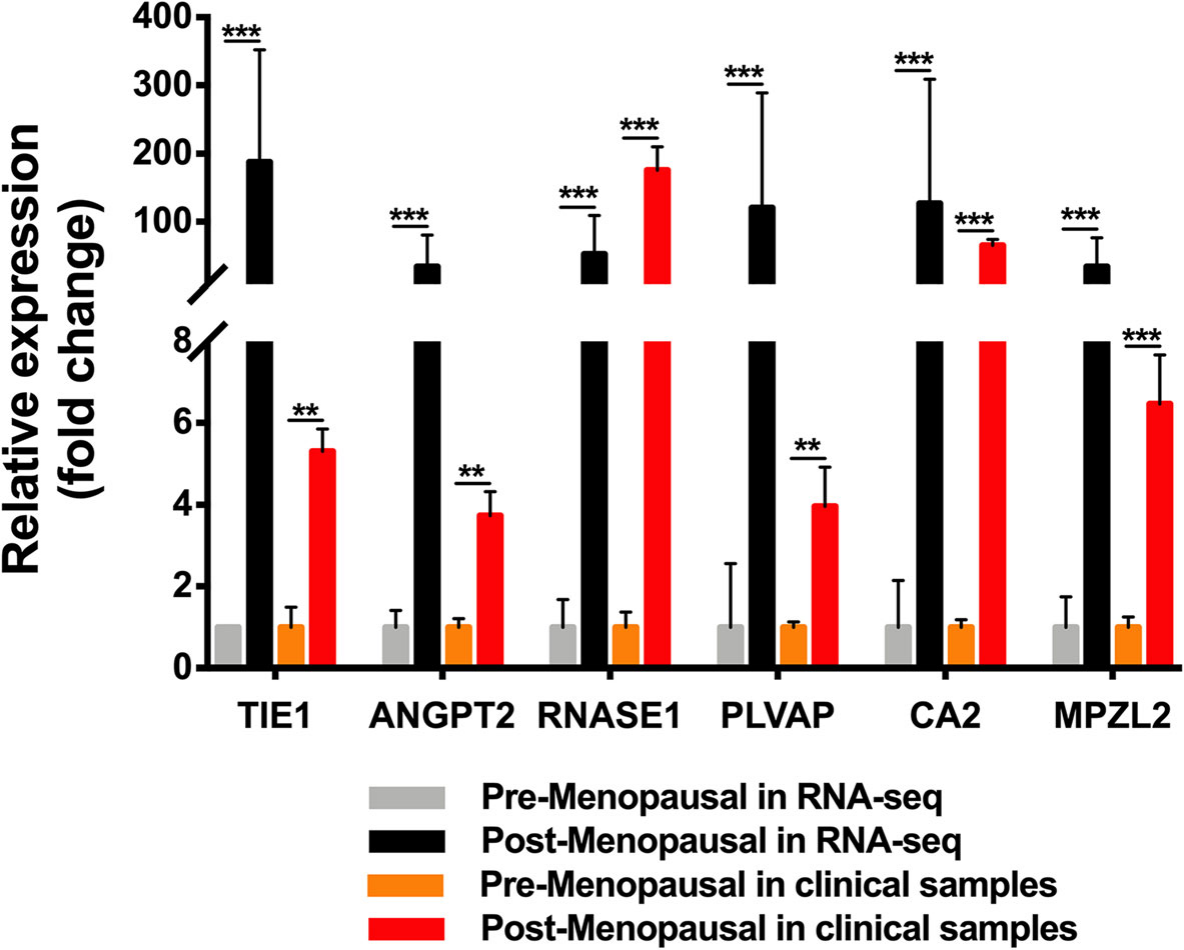
Confirmation of hub gene expression. The relative expression of TIE1, ANGPT2, RNASE1, PLVAP, CA2, and MPZL2 in RNA-seq and clinical samples. *p < 0.05, **p < 0.01, and ***p < 0.001 vs premenopausal RNA-seq or clinical samples. The expression profile of the top hub genes agreed between the RNA sequencing data and clinical samples

hASCs were isolated and confirmed positive for CD73, CD90, and CD105 and negative for CD34, CD45, and HLA-DR according to flow cytometry analysis of stromal cell-related surface markers (Supplementary File 8). The ability of the generated hASC cultures to differentiate into multiple cell types was confirmed by using oil red O staining for lipid droplet formation, alizarin red S staining for calcium deposit formation, and Alcian blue staining for sulfated proteoglycan formation (Supplementary File 9). The expression of the top hub genes, including TIE1, ANGPT2, RNASE1, PLVAP, CA2, and MPZL2, was verified by RT-PCR. The postmenopausal clinical sample group possessed higher expression levels of TIE1, ANGPT2, RNASE1, PLVAP, CA2, and MPZL2 (Fig. 4), which agreed with the RNA sequencing data.

### Immunoregulatory role of ASCs from pre- and postmenopausal women

**Fig. 5:**
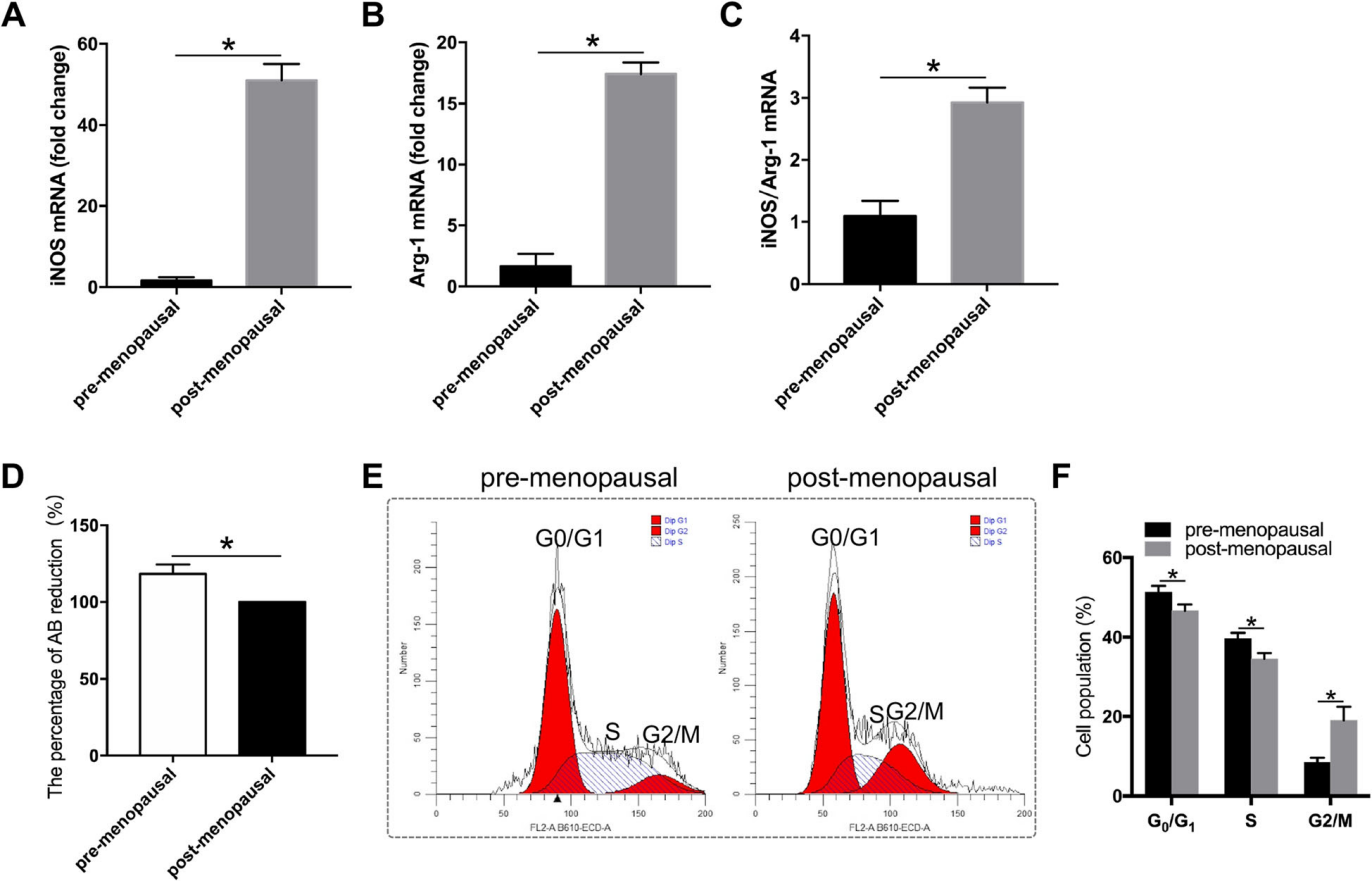
Immunoregulatory role of ASCs from pre- and postmenopausal women. **a** Relative mRNA expression of iNOS. *p < 0.05 vs premenopausal. **b** Relative mRNA expression of Arg-1. *p < 0.05 vs premenopausal. **c** The ratio of mRNA expression of iNOS and Arg-1. *p < 0.05 vs premenopausal. **d** Metabolic activity was analyzed by the Alamar Blue assay. *p < 0.05 vs premenopausal. **e** The cell cycle was analyzed by flow cytometry. **f** Quantitative analysis of the cell population in G_0_/G_1_ phase, S phase, and G_2_/M phase of the cell cycle. *p < 0.05 vs premenopausal

THP-1 cells co-cultured with ASCs derived from postmenopausal women expressed more iNOS and less Arg-1 than their counterparts (Fig. 5a–c), indicating that ASCs derived from postmenopausal women are inclined to elicit macrophage M1 polarization. CD3/4+ T cells co-cultured with ASCs derived from premenopausal women exhibited increased proliferation activity (Fig. 5d), while those co-cultured with ASCs derived from postmenopausal women showed G_2_/M arrest (Fig. 5e, f).

## Discussion

As the population ages, postmenopausal women are becoming a more important source of ASCs, and there is an increasing demand for ASC-related treatments for postmenopausal women. Therefore, the transcriptome differences between ASCs derived from pre- and postmenopausal women should be identified. The present study identified DEGs between pre- and postmenopausal women and applied integrated annotation methods to annotate them. We also applied two gene coexpression network analysis approaches to identify gene networks and hub genes from ASCs derived from pre- and postmenopausal women. A holistic view was generated by preserving gene interactions, which helps deepen our understanding of ASCs.

Pre- and postmenopausal women were clustered into two clusters in the DEG hierarchical cluster analysis, indicating that ASCs derived from pre- and postmenopausal women may be quite different at the transcriptome level. GO and pathway analyses provided a snapshot of the genetic variance between pre- and postmenopausal women and revealed some fertility-, sex hormone-, immune-, aging-, and angiogenesis-related terms or pathways. Pathway analysis was applied to the yellow module and multiscale hub genes and found that they share immune-, angiogenesis-, and sex hormone-related pathways. We also noticed a high consistency among the top hub genes in the yellow module and the multiscale hub genes in MEGCN. These top hub genes included TIE1, ANGPT2, RNASE1, PLVAP, CA2, and MPZL2. Their consistency could support the authenticity of WGCNA and MEGENA to some extent. The expression of some top hub genes has also been verified with clinical samples.

In addition to pure bioinformatics analysis, we also performed functional assays to identify the immunoregulation difference in ASCs from pre- and postmenopausal women. We found that ASCs from postmenopausal women could induce M1 polarization of macrophages, while their counterparts facilitate CD3/4+ T cell proliferation. These results are consistent with our GO and pathway analysis. However, these results are quite preliminary, and more work is needed to determine the mechanism.

Unlike bone marrow mesenchymal stromal cells [12], the relationships between ASCs and menopausal status have never before been researched. Although some studies have investigated the role of aging and sex hormones on ASCs, the present study is different from them. Several studies have determined the negative role of age on ASCs, such as those described by Monika et al. [38] and Ma et al. [39]. These studies not only deepen our understanding of ASCs but also instruct the clinical application of ASCs. Regrettably, their aging criteria are inconsistent, and this is a major problem that similar studies have faced. Menopause, to some extent, could be seen as aging with explicit criteria, which is the reason many aging-related results were identified in the present study. For sex hormones, the situation is similar. The present study found that ASCs derived from pre- and postmenopausal women are not equivalent, at least not at the transcriptome level. Thus, more attention should be paid when applying ASCs derived from postmenopausal women. Moreover, approaches to enhance the function of ASCs may also be needed.

Liang et al. proposed that genetic modification of pivotal genes might alter the paracrine profile and maximize the therapeutic potential of mesenchymal stromal cells [40]. Many preconditioning approaches have been applied to improve the function of ASCs, such as epidermal growth factor and basic fibroblast growth factor [41], vascular endothelial growth factor (VEGF) [42], and many other factors [43]. The top hub genes identified in the present study could serve as potential targets, as they are not only key players in the gene coexpression network but also have important functions.

Some top hub genes may affect vascular homeostasis. TIE1 [44], ANGPT2 [45], and PLVAP [46] all interact with VEGF. TIE1 was reported to control angiogenesis and vascular remodeling [47, 48]. Failure to establish the structural integrity of vascular endothelial cells has been found in embryos deficient in TIE1 [49]. ANGPT2 was reported to promote angiogenesis by destabilizing blood vessels under the regulation of VEGF [50, 51]. Specifically, ANGPT2 is associated with follicular growth and angiogenesis during the preovulatory period in premenopausal women [52]. PLVAP could regulate angiogenesis and vascular permeability [53, 54]. RNASE1, though not specifically related to VEGF yet, encodes a member of the ribonuclease A superfamily, which is an important member in angiogenin [55]. As extracellular RNA in the vascular system can enhance coagulation and permeability, RNASE1 could maintain vascular homeostasis by regulating extracellular RNA [56].

Some top hub genes may also participate in immunomodulation. ANGPT2 can recruit monocytes [57, 58], which results in suppressing T cell activation [59]. MPZL2 could regulate the development of the thymus and T cell function [60, 61]. As mentioned above, PLVAP can regulate vascular permeability. Thus, it is not surprising that PLVAP was reported to mediate leukocyte transendothelial migration [62].

In addition to vascular homeostasis and immunomodulation, these top hub genes also have other related functions. ANGPT2 can enhance osteogenic differentiation and angiogenesis in bone marrow stem cells [63]. Therefore, alteration of the ANGPT2 expression profile may affect ASC differentiation. MPZL2 was found to maintain the pluripotency and self-renewal of glioblastoma-initiating cells [64]. Therefore, MPZL2 could play a role in maintaining the stemness of ASCs. CA2 is regulated by estrogen and androgens [65, 66] and plays a major role in osteoclast differentiation and bone resorption [67]. Zheng et al. found that ovariectomy results in the upregulation of CA2, which could be reversed by administration of 17 beta-estradiol [67]. They proposed that CA2 may be a target of estrogen’s protective role in bone loss. Therefore, upregulation of CA2 in ASCs may participate in bone loss in postmenopausal women, and targeting CA2 may improve the function of ASCs harvested from postmenopausal women.

One limitation of the present study is that all ASCs were isolated from adipose tissue harvested by abdominoplasty. ASCs from different donor sites may have different characteristics. For example, Cox-York and her colleagues found that estradiol, a type of estrogen, has region-specific effects on ASCs in postmenopausal women [68]. Therefore, ASCs from other anatomical sites may have different transcriptomic landscapes in postmenopausal women. However, menopause promotes systematic and extensive changes such as metabolic abnormalities and body fat re-distribution [69, 70]. Consequently, transcriptomic differences in ASCs at other anatomic sites are expected.

## Conclusions

In conclusion, the present study revealed the transcriptome differences in adipose stem cells derived from pre- and postmenopausal women and provides holistic views by preserving gene interactions with gene coexpression network analyses. The top hub genes identified by the present study could serve as potential targets to enhance the therapeutic potential of ASCs.

## Supplementary information

**Additional file 1.** A complete list of the identified genes differentially expressed in the pre- and postmenopausal women.

**Additional file 2.** The complete hierarchical cluster results of pre- and postmenopausal women.

**Additional file 3.** The slim term Ageing identified among the downregulated DEGs.

**Additional file 4.** Complete pathway analysis results.

**Additional file 5.** A summary of the WGCN.

**Additional file 6.** The complete MEGCN.

**Additional file 7.** Primers used in this study.

**Additional file 8.** Immunophenotypic analysis of ASCs.

**Additional file 9.** Multipotential induction of ASCs.

## Data Availability

The datasets supporting the conclusions of this article are included within the article (and its additional files).

## Abbreviations

ANGPT2: Angiopoietin 2
ASCs: Adipose stromal cells
CA2: Carbonic anhydrase 2
DEGs: Differentially expressed genes
GAPDH: Glyceraldehyde-3-phosphate dehydrogenase
GO: Gene Ontology
KEGG: Kyoto Encyclopedia of Genes and Genomes
MEGCN: Multiscale Embedded Gene Coexpression Network
MEGENA: Multiscale Embedded Gene Coexpression Network Analysis
MPZL2: Myelin protein zero-like 2
NCBI: National Center for Biotechnology Information
PFN: Planar Filtered Network
PLVAP: Plasmalemma vesicle-associated protein
RNASE1: Ribonuclease A family member 1
RT-PCR: Real-time polymerase chain reaction
TIE1: Tyrosine kinase with immunoglobulin-like and EGF-like domains 1
WGCN: Weighted Gene Coexpression Network
WGCNA: Weighted Gene Coexpression Network Analysis
