# Supplementary figures and images for: Transcriptome differences in adipose stromal cells derived from pre- and postmenopausal women
Source: Stem Cell Res Ther. 2020 Feb 28;11:92. doi: 10.1186/s13287-020-01613-x (PMC7049195; doi:10.1186/s13287-020-01613-x)

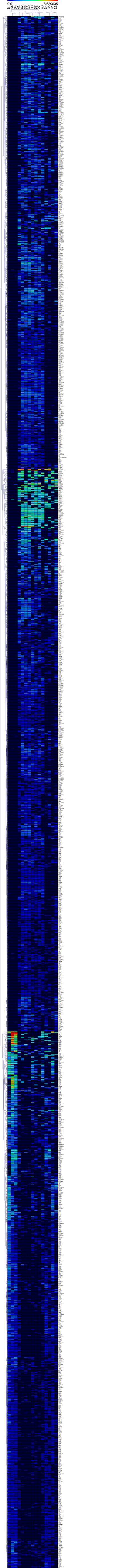

Supplement: Supplementary file 2 — Additional file 2. The complete hierarchical cluster results of pre- and postmenopausal women. [file 13287_2020_1613_MOESM2_ESM.tif]

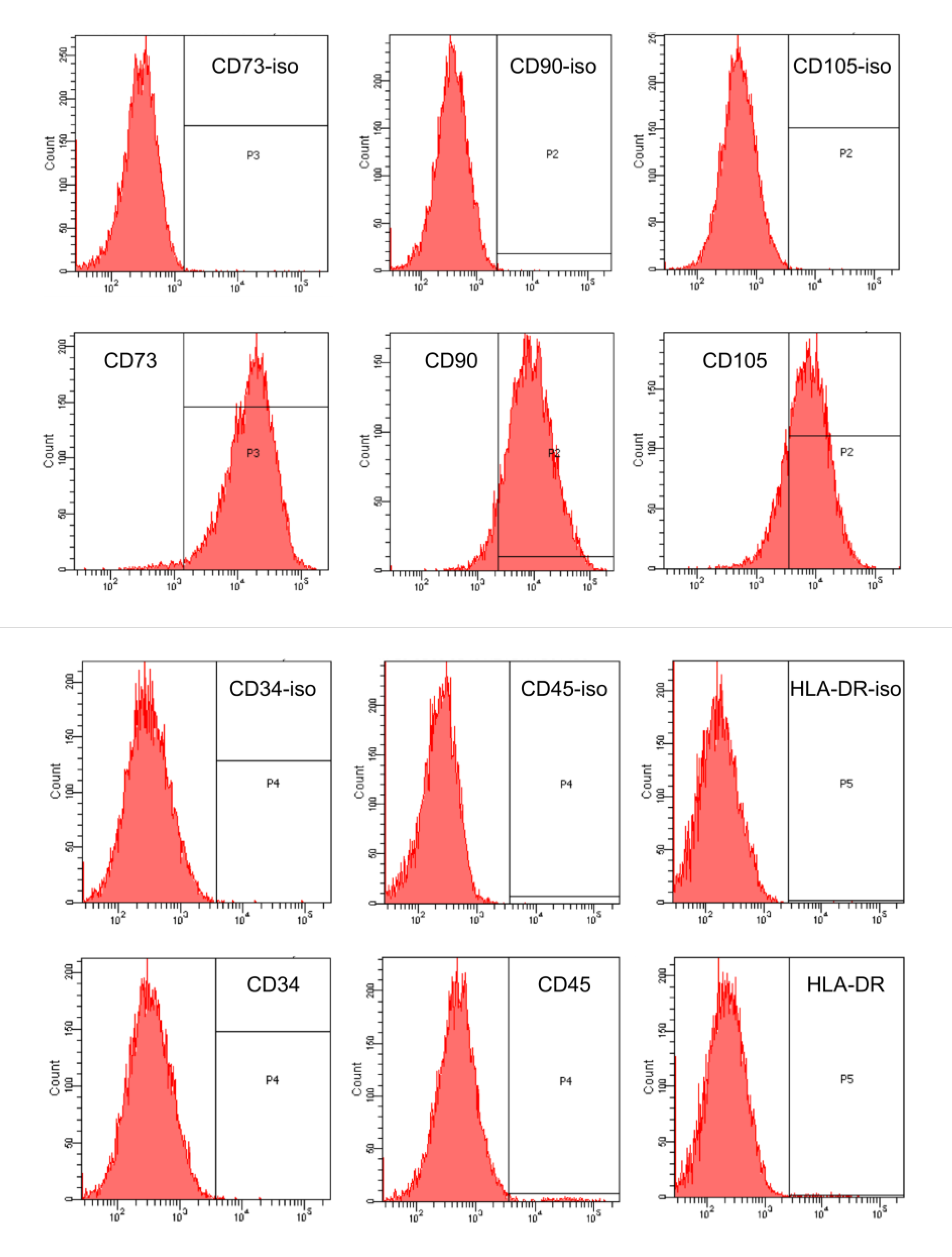

Supplement: Supplementary file 8 — Additional file 8. Immunophenotypic analysis of ASCs. [file 13287_2020_1613_MOESM8_ESM.tif]

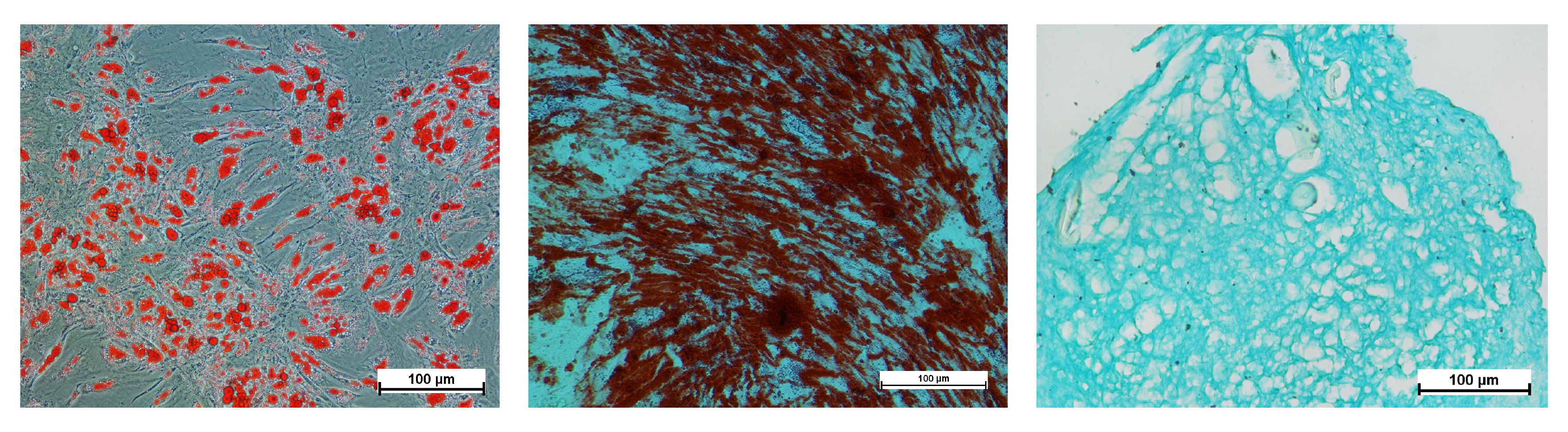

Supplement: Supplementary file 9 — Additional file 9. Multipotential induction of ASCs. [file 13287_2020_1613_MOESM9_ESM.tif]
